# Description of Fifteen New Species of Crinoidea from the Sub-carboniferous Limestone of Iowa, Collected during the United States Geological Survey of Iowa, Wisconsin, and Minnesota, in the Years 1848–’9

**Published:** 1851-09

**Authors:** 


ART. VII. — *Description of fifteen new Species of Crinoidea from the Sub-Carboniferous Limestone of Iowa, collected during the United States Geological Survey of Iowa, Wisconsin, and Minnesota, in the years* 1848–'9. By DAVID D. OWEN, M.D., and B. F. SHUMARD, M. D. Journal of the Academy of Natural Sciences of Philadelphia. New Series. Vol. II. Part I. Philadelphia. 1850.

The study of Fossils appears to most persons one of the least inviting of all the pursuits in which scientific men engage. It is looked upon by many as not only dry and unattractive, but absolutely trivial and unworthy of the attention of great and serious minds. Polypes and mollusks, the bones of fishes, reptiles, and birds, what is there in these, it is sometimes asked, to repay one for their investigation? What can one learn that is worth knowing, by groping among the remains of the humblest tenants of a former world—the corals, trilobites, and crinoids imbedded in the rocks?

In themselves, these organic remains, at first sight appear, indeed, to have little to interest us. They relate to tribes of animals of the simplest structure, the lowest down in the animal scale, which have generally passed away, many of them leaving no representatives behind them. They are the farthest possible removed from the human species, and the lowest of them seem to be as closely allied to the vegetable as to the animal races. Nevertheless, the study of their organization and functions, their habits and distribution, has brought out results highly interesting to the physiologist and of the utmost importance in geology. Let us illustrate a little. These inferior tribes of the animal kingdom constitute the zero of the physiologist—his starting point in determining the functions of the various parts of the animal system; and it is only by running his eye along the whole chain of being that he can gain an insight into the economy of the higher races. One of the most startling of the modern discoveries in physiology is the fact, that these higher races commence existence in forms allied to the lower—that at one period the embryo mammal resembles a bird, at an earlier state, a reptile, and at one still earlier, a fish. All animals alike begin as ova; the embryo of the highest is scarcely removed at first from the molluscous animal, and advances through the successive stages up to its matured organization. It is not true that the quadruped is at any stage of its being a bird, a fish, or a reptile, but its resemblance in its immature state to these families shows that their position is a lower one in the animal scale. And in like manner we are able to determine the relative grade of the several species of a family. Thus, those birds which are web-footed only during the first days of embyronic life rank higher in the scale than those in which this character remains permanent. The duck family are of an inferior grade to thrushes, pigeons, and birds of prey.

Now, the principle taught by embryology is the same which we derive from the study of fossils. The order of succession among animals in our geological formations is the order observed in the development of the embryo. In the lowest and oldest rocks, we find the remains only of the humblest tribes of beings—the mollusca, trilobites, and encrinites. As we ascend, we encounter the petrified teeth and scales of fishes; then we reach the bones of reptiles; next we find those of birds; and finally, mingling in the later beds, we meet with the skeletons of mammalia.

From this brief illustration it will be perceived that paleontology—the science of the extinct tenants of our globe—is not without its bearing upon at least one branch of medicine. Physiology is interested in the progress of geological science. It is deriving valuable truths from the investigation of the structure of those races which, having swarmed in our primitive seas, are now only known by their solid parts that have hardened into stone; and some of the most interesting principles of the science are deriving confirmation from the discoveries of the geologist.

In the prosecution of geology, a knowledge of organic remains is indispensable. It is by these that we determine the succession of strata; they are the "medals of creation"—the characters inscribed on the rocks which inform us of the history of our planet. One of these fossils, apparently unmeaning and valueless—to the uneducated eye as meaningless as the pebble by the brook-side—is full of significance to the man of science. It speaks to him of a specific formation and of a particular age. By it he is enabled to decide on the relative position of the strata from which it came, and to compare them with the strata of other countries. Thus, for example, one of the Encrinites described by Drs. Owen and Shumard, in the hands of a European geologist, is a key to the geology of our country. He needs only to see one of these organisms to decide whether the rocks in which it was entombed belong to one or another of the formations of Europe. We ascertain our geological position by means of these remains. And thus it turns out that trivial as they at first appear, they come to have a wonderful interest when regarded as the characters by which the history of our globe is to be decyphered. The medals and coins of a country —the old blackletter and other styles of printing, the cotton-gin, the steam-engine, and the electrical telegraph —are not more characteristic of a particular period in human history than are these fossils of a particular era in creation. The trilobite marks one age; the ammonite is peculiar to a later period; nor is the order of their appearance ever reversed. The geologist knows with certainty that the strata which contain the latter are of a later age than those containing the former. When he exhumes the remains of a reptile he is sure that he is not among the rocks of the earliest era; and when he comes upon the fossil bones of mammals, he knows that he is among strata deposited just before the dawn of the existing races. In this view, the remains of these by-gone species may be said to be the basis upon which geology as a science reposes. The geologist can make no progress without a knowledge of fossils.

As different species of animals are now found existing in different countries, so among the remains of extinct species we find some abounding in one portion and others in another quarter of the earth. Some are common to two continents; a few families have survived all the revolutions of our planet, and have had their representatives in every era. The trilobites especially abound in Europe; our continent surpasses all othe countries in the number and variety of our encrinites.

Dr. Shumard and the writer, although they have been but a few years engaged in collecting these beautiful fossils, have already in their cabinet twice as many species as are known to the geologists of Europe. The late Dr. Troost has left behind him a monograph on the Crinoidea of Tennessee, in which he has described more than a hundred species, nearly all of which are new.

It is a curious and interesting fact, that scarcely a single species of Encrinite discovered in the Western

States has hitherto been found in Europe. Of our two hundred species, we incline to believe that not a single one is common to Europe and our country.

Another striking fact is, that each species is confined strictly to a single geological formation. Dividing our rocks into those of the Silurian, Devonian, and Carboniferous periods, we find no crinoid ranging through the three, nor even extending from one into the other. The Silurian encrinites are marked by peculiar features, by which they are readily distinguished from those of the Devonian era, and these again differ widely from those of the Carboniferous period. A family is seen in the older Silurian rocks, but as we ascend in the series it disappears and is succeeded by a family of a different type. This again gives place to genera of another form, and these, in turn, to other genera as we pass through the successive series. The eye of a practiced geologist no sooner rests upon a crinoid than he decides upon its geological position. The family features have been preserved, but the type has changed in successive ages, and the encrinite of the latest formations is a very different animal from that which flourished amid primeval seas.

These animals are extremely local in their distribution and have a limited geographical range. While many other genera are widely diffused, the remains of the crinoidea are found only in a few circumscribed spots. But few species are common to New York and the Western States; a small number range over three or four States, but far the greater number have been discovered at a single locality. They seem to have demanded for their growth and development peculiar conditions, which were afforded by comparatively few parts of the ocean. They probably inhabited shallow water and were affected by slight changes of climate, which circumstance would necessarily impose upon them a circumscribed range. Many of the mollusca, which live in deep water, extended from New York to Russia, while, as we have seen, probably not a single species of encrinite was a common tenant of the two continents.

The crinoidea appear to have been carnivorous in their habits, many of the specimens in our cabinet having shells enclosed in their arms, as if the animal had been in the act of feeding when it perished.

But this is not the place for a minute history of the habits of this singular tribe of beings; we fear we have already said more respecting them than will be interesting to our readers. But some notice of the subject seemed to be due to our brethren, the authors of the monograph which stands at the head of this article, who are devoting their time and energies to the advancement of natural science in our country. This paper contains the first description yet given of any of these fossil species in which, as we have already remarked, the Western States abound beyond any other quarter of the globe. We will only add, that it is a creditable performance. The descriptions are clear, accurate, and full, and the figures, though not in the best style of American art, are well executed. In the first particular, the work of our Western geologists will compare favorably with the works of that accomplished paleontologist, Jas. Hall, Esq., of New York; in the beauty and fidelity of the figures it is not equal to the volumes issued under his care. It is proper to add that while the credit of the letter-press belongs to the authors of the monograph, the responsibility of the figures rests with the engraver. Y.

